# Deep sequencing shows microRNA involvement in bovine mammary gland adaptation to diets supplemented with linseed oil or safflower oil

**DOI:** 10.1186/s12864-015-1965-7

**Published:** 2015-10-30

**Authors:** Ran Li, Frédéric Beaudoin, Adolf A. Ammah, Nathalie Bissonnette, Chaouki Benchaar, Xin Zhao, Chuzhao Lei, Eveline M. Ibeagha-Awemu

**Affiliations:** Agriculture and Agri-Food Canada, Dairy and Swine Research and Development Centre, Sherbrooke, Quebec J1M 0C8 Canada; College of Animal Science and Technology, Northwest A&F University, Xi’an, Shaanxi 712100 China; Department of Animal Science, McGill University, Ste-Anne-de-Bellevue, Quebec H9X 3V9 Canada

**Keywords:** microRNA, Bovine mammary gland, Linseed oil, Safflower oil, Lipogenesis

## Abstract

**Background:**

Bovine milk fat composition is responsive to dietary manipulation providing an avenue to modify the content of fatty acids and especially some specific unsaturated fatty acid (USFA) isomers of benefit to human health. MicroRNAs (miRNAs) regulate gene expression but their specific roles in bovine mammary gland lipogenesis are unclear. The objective of this study was to determine the expression pattern of miRNAs following mammary gland adaptation to dietary supplementation with 5 % linseed or safflower oil using next generation RNA-sequencing.

**Methods:**

Twenty-four Canadian Holstein dairy cows (twelve per treatment) in mid lactation were fed a control diet (total mixed ration of corn:grass silages) for 28 days followed by a treatment period (control diet supplemented with 5 % linseed or safflower oil) of 28 days. Milk samples were collected weekly for fat and individual fatty acid determination. RNA from mammary gland biopsies harvested on day-14 (control period) and on days +7 and +28 (treatment period) from six randomly selected cows per treatment was subjected to small RNA sequencing.

**Results:**

Milk fat percentage decreased significantly (*P* < 0.001) during treatment with the two diets as compared to the control period. The individual saturated fatty acids C4:0, C6:0, C8:0, C14:0 and C16:0 decreased significantly (*P* < 0.05) while five USFAs (C14:1, C18:1n11t, C20:3n3, C20:5n3 and CLA:t10c12) increased remarkably (*P* < 0.05) in response to both treatments. Analysis of 361 million sequence reads generated 321 known bovine miRNAs and 176 novel miRNAs. The expression of fourteen and twenty-two miRNAs was affected (*P* < 0.05) by linseed and safflower oil treatments, respectively. Seven miRNAs including six up-regulated (bta-miR-199c, miR-199a-3p, miR-98, miR-378, miR-148b and miR-21-5p) and one down-regulated (bta-miR-200a) were found to be regulated (*P* < 0.05) by both treatments, and thus considered core differentially expressed (DE) miRNAs. The gene targets of core DE miRNAs have functions related to gene expression and general cellular metabolism (*P* < 0.05) and are enriched in four pathways of lipid metabolism (3-phosphoinositide biosynthesis, 3-phosphoinositide degradation, D-myo-inisitol-5-phosphate metabolism and the superpathway of inositol phosphate compounds).

**Conclusion:**

Our results suggest that DE miRNAs in this study might be important regulators of bovine mammary lipogenesis and metabolism. The novel miRNAs identified in this study will further enrich the bovine miRNome repertoire and contribute to understanding mammary gland biology.

**Electronic supplementary material:**

The online version of this article (doi:10.1186/s12864-015-1965-7) contains supplementary material, which is available to authorized users.

## Background

Milk fat determines the physical properties and quality of milk and milk products and is also the main energy source in whole milk [1]. Bovine milk fat typically consists of 70 % saturated fatty acids (SFAs) which could exert negative effects when consumed in excess, as well as 25 % monounsaturated fatty acids (MUFAs) and 5 % polyunsaturated fatty acids (PUFAs) both of which have potential positive effects on human health [2, 3].

In ruminants, milk fat has been found to be especially responsive to dietary manipulation [4], providing an avenue to modify the fatty acid (FA) profile of milk fat in favor of health promoting isomers, and also a great model for scientists to study the mechanisms of mammary lipogenesis. Dietary supplementation with plant oils that are rich in unsaturated fatty acids (USFAs) can lead to significant reductions in milk fat yield of up to 50 % [5] as well as increased concentrations of conjugated linoleic acids (CLA) in bovine milk [6]. Therefore, supplementation of cow diets with USFAs from plant oils has been considered as a feasible dietary strategy to improve beneficial FA contents of milk and milk products [7]. Linseed oil (rich in α-linolenic acid, about 57 % of total fat, C18:3n3) is one of the typical USFA-rich plant oils that are frequently used as a dietary supplement to manipulate milk FA composition. Numerous studies have shown that dietary supplementation with linseed oil can reduce milk fat yield through repression of *de novo* FA synthesis, resulting in increased milk CLA, omega-3 FA levels as well as other USFAs [8–11]. Safflower oil/seed (rich in linoleic acid, about 76 % of total fat, C18:2n6) supplementation can also affect FA composition of ruminant milk, resulting in increased concentrations of CLA in bovine milk [6, 12] and in lean tissue of sheep [13]. Recently, it was found that addition of 3 % safflower oil to the diet can induce a decrease in milk fat yield, short-chain FA yield and C16:0 yield and an increase in milk C18:1n10t and CLA:10t12c yield [14].

Milk fat is derived from *de novo* synthesized FAs by mammary epithelial cells and preformed FAs taken up from blood circulation [15]. Milk fat synthesis requires the coordinated regulation of enzymatic activities in pathways of metabolite transport, *de novo* lipogenesis, FA transport, desaturation, and esterification, and milk fat globule formation and secretion [16]. Milk fat synthesis is driven by a coordinated participation of key lipogenic enzymes. For example, stearoyl-CoA desaturase 1 (SCD1) is responsible for MUFA synthesis, while FA desaturases (FADS1, FADS2 and FADS3) are involved in synthesis of long chain FAs [17]. Long term dietary supplementation with a mixture of linseed oil and algae resulted in down-regulation of SCD1, FA synthase (FASN) and the regulatory element binding transcription factor (SREBF1) [18]. Abomasal infusion with linseed oil also affected expression of lipogenic genes in the bovine mammary gland, including decreased abundance of SCD [19]. However, the molecular mechanism by which dietary USFAs alter transcription activities of lipogenic genes and thus affect the pathways of lipogenesis as well as individual FA profiles is not well established.

MicroRNAs (miRNAs) are small regulatory RNA molecules that have been shown to be involved in a wide range of biological pathways by modulating expression of specific mRNAs [20]. The role of miRNAs in lipid metabolism has been recently demonstrated [21] and lipid homeostasis was found to be governed in part by an intricate web of miRNA activity [22]. In bovine mammary gland, the expression of numerous miRNAs was increased during the postpartum or the early lactation periods as compared to the dry period [23]. Comparative transcriptome profiling using high throughput sequencing has also revealed considerable differentially expressed miRNAs between lactating and non-lactating bovine mammary glands [24] thus suggesting a critical role of miRNAs in mammary gland development and lactation. Even the overexpression of one miRNA (miR-30d) could cause dysregulation of lactation and delay mammary gland involution in mouse [25]. Another study found that ten miRNAs in early lactation could be essential in mammary lipid biosynthesis of rats by putatively targeting down-regulated genes expressed in the mammary gland [19]. In dairy goats, miR-27a and miR-103 have been found to function as regulators of milk fat metabolism and lactation cycle [26, 27]. Furthermore, studies have found that miRNA expression profile can be manipulated by dietary fat composition in adipose tissues of lambs [28] and cattle [29]. Thus, we suppose that miRNAs could play critical roles in milk fat synthesis in bovine mammary glands and contribute to the process of milk fat depression as well as increased milk beneficial FAs in response to diets rich in USFAs.

In order to explore the potential role of miRNAs in mammary lipogenesis, miRNA expression profiles of bovine mammary glands from cows fed diets supplemented with 5 % (on dry matter bases) linseed oil (6 cows) or safflower oil (6 cows) were investigated using next generation small RNA sequencing technology. The high throughput sequencing data enabled us to examine the bovine mammary gland miRNome profile and uncovered differentially expressed miRNAs in response to the dietary treatments, which will help further understanding of miRNA involvement in bovine mammary gland lipogenesis.

## Methods

### Ethics statement

All the experimental procedures were approved by the Animal Care and Ethics Committee of Agriculture and Agri-Food Canada.

### Animals, diets and samples collection

Twenty-four high producing Canadian Holstein cows in mid-lactation were blocked according to parity and days in milk and randomly assigned to one of two treatments (12 cows/treatment), namely linseed oil treatment (rich in α-linolenic acid, C18:3n3, 57 % of total fat) and safflower oil treatment (rich in linoleic acid, C18:2n6, 76 % of total fat) (Additional file 1). The experiment started with a control period of 28 days during which time the diet consisted of total mixed ration of corn:grass silages (50:50) and concentrates (control diet). This was followed by a period of supplementation of 28 days (treatment period) during which cows were fed the control diet supplemented with 5 % linseed oil (DM) or 5 % safflower oil. Mammary gland biopsies were collected from 12 cows (6/treatment, randomly selected) at three different time points during the experiment: day-14 (middle of control period), day + 7 (seven days after onset of treatment), and day + 28 (28 days after onset of treatment) following an established protocol [30]. Biopsy was performed on the same quarter and approximately the same site each time. About 400 mg of tissue (70 x 4 mm in diameter) was collected from each cow per biopsy, snap-frozen in liquid nitrogen and stored at −80 °C until isolation of total RNA.

Milk samples were collected from cows weekly (day-14, day-7, day-1, day + 7, day + 14, day + 21 and day + 28). At each sampling, a volume of 40 ml of milk from each cow was collected at morning and evening milking and thoroughly mixed to get a composite sample. One portion of the composite milk was sent to Valacta (St-Anne-de-Bellevue, Quebec, www.valacta.com) for determination of test day milk fat percentage. The other portion was used for determination of individual FA composition. Fatty acid methyl esters (FAME) were prepared from milk fat according to O’Fallon *et al*. [31]. FA composition of milk fat was determined using Agilent 6890 N gas chromatograph (Agilent Technologies, USA) equipped with flame ionisation detector.

### Statistical analysis

The response of test day milk fat percentage and individual FAs to treatments was analyzed with SAS software (version 9.3, SAS Institute Inc., USA). A completely randomized design with repeated measures and mixed effects ANOVA model (F-test) with Tukey adjustment was used to determine the effects of treatments on test day milk fat percentage and FA composition by comparing early treatment period (day + 7) vs. control (day-14), late treatment period (day + 28) vs. control and late treatment period vs. early treatment period. For some FAs, the data was asymmetrically distributed and thus log transformed to fit a normal distribution before applying the ANOVA model. The mean values of the log transformed data were back transformed with a 95 % confidence interval for result interpretation.

### RNA extraction

Total RNA from mammary gland biopsy (50 mg/sample) at day-14 (control period), day + 7 and day + 28 (treatment period) were purified using miRNeasy Kit (Qiagen Sciences, USA) following manufacturer’s instructions. The quantity and quality of purified RNA were respectively determined using NanoDrop 1000 (NanoDrop Technologies, USA) and Agilent 2100 Bioanalyzer (Agilent Technologies, USA). RNA samples for sequencing had RIN (RNA integrity number) values from 7.5 to 9.0.

### miRNA library preparation and sequencing

A total of thirty-six libraries were prepared and barcoded for sequencing according to a previous protocol [32] with minor modifications. Briefly, total RNA was first ligated to a primer at the 3’end (3’adaptor) by T4 RNA Ligase 22tr K227Q (New England Biolabs Inc., Canada) which was then annealed to a reverse transcription primer to prevent undesirable dimerization of 3’ and 5’ adaptors in the following step. Before reverse transcription, the 5’adaptor was ligated to the 5’end of the RNA by T4 RNA Ligase 1 (Enzymatics Inc., USA). This RNA:DNA hybrid was reverse transcribed into cDNA using Superscript III Kit (Life Technologies, USA). The barcoding of the different libraries was finally achieved by PCR using primers which consisted of specific barcodes for each library. Size separation of the miRNA libraries was performed by polyacrylamide gel electrophoresis. The libraries were then eluted from the gel using an elution buffer (10 mM Tris–HCl pH 7.5; 50 mM NaCl, 1 mM EDTA) and concentrated using DNA clean and concentrator-5 from Zymo Research (Zymo Research, USA). Finally, the concentration of the purified libraries was evaluated by Picogreen assay (Life Technologies) on a Nanodrop 3300 fluorescent spectrophotometer.

Multiplexed libraries (12 libraries per lane) were subjected to 50 bp single end sequencing on an Illumina HiSeq 2000 system (Illumina Inc. USA) by McGill University and Genome Quebec Innovation Centre (Montreal, QC, Canada) using TruSeq v3 (Illumina Inc.) reagents. Barcodes (indexes) and adaptor sequences for multiplexed samples are shown in additional file 2.

### Small RNA sequence data analysis

The raw data of 36 fastq files were checked for sequencing quality with FastQC program version 0.10.1 (http://www.bioinformatics.babraham.ac.uk/projects/fastqc/). Cutadapt v1.2.2 (http://code.google.com/p/cutadapt/) was used to trim 3’ adaptor sequences and filter 5’ adaptor contaminants and repeats. Reads shorter than 18 nucleotides after trimming or having a low Phred score of less than 20 for at least 50 % of the bases were discarded. Low quality bases with Phred score <20 corresponding to a probability of error higher than 0.01 [33] as well as reads containing unknown bases were removed using FASTQ Quality Filter tool of FASTX-toolkit (http://hannonlab.cshl.edu/fastx_toolkit/). For novel miRNA discovery, clean reads from the 35 files that passed all filtering criteria were parsed into one file and mapped to the bovine genome (Bta_4.6.1) using bowtie 1.0.0 [34]. Reads that mapped to more than five positions of the genome were discarded. Furthermore, reads that mapped to bovine rRNA, tRNA, snRNA and snoRNA in the Rfam RNA family database (http://rfam.sanger.ac.uk/) were annotated using blastn of blast + (v2.2.28) package [35].

### Identification of known miRNA and novel miRNA discovery

The identification of known miRNA and discovery of novel miRNA were performed using miRDeep2 v2.0.0.5 [36] which uses a probabilistic algorithm based on the miRNA biogenesis model and designed to detect miRNAs from deep sequencing reads. The core module of miRDeep2 was used to identify known miRNA and novel miRNA in the pooled dataset of all the libraries. The Quantifier module of miRDeep2 was used to profile the detected miRNAs in each library. The miRDeep2 software outputs a scored list of known and novel miRNAs with log-odds score to help in the determination of true known and novel miRNAs. In this study, only miRNAs with a greater than one count per million (CPM) in at least ten libraries were considered as true known miRNAs. In order to predict novel miRNAs with high confidence, only those with a miRDeep2 score higher than five and present in at least three libraries with at least 10 total counts were retained as true novel miRNAs. The other potential novel miRNAs that didn’t meet all the above criteria but with a miRDeep2 score higher than five or at least 100 reads were classified as novel miRNA candidates. Possible modifications within miRNA sequences were analyzed with Chimira v1 program [37] (http://wwwdev.ebi.ac.uk/enright-srv/chimira/about.php).

### Differential expression analysis

The R (v3.0.1) package edgeR (v2.4.6) [38] which uses a negative binomial model was used to identify significantly differentially expressed miRNAs (including known and novel miRNAs) in response to the treatments. The trimmed mean of Mvalues (TMM) normalization method, which assumes that the majority of the genes are not differentially expressed [39], was used for normalization of the miRNA expression count data. Significantly differentially expressed miRNAs were defined as having a Benjamini and Hochberg [40] corrected *P* value ≤ 0.05.

### Function analysis of significantly differentially expressed miRNAs

The target genes of DE miRNAs were predicted using Ingenuity Pathway Analysis (IPA) software (Ingenuity Systems Inc., USA). MiRNA target prediction information in IPA database is very comprehensive as it includes not only bioinformatics predictions using TargetScan (www.targetscan.org), but also from experimentally validated information on gene-miRNA interactions from TarBase database (http://www.microrna.gr/tarbase) and miRecords (http://c1.accurascience.com/miRecords/). The miRNA target filter function of IPA enabled us to focus on the target genes that were experimentally observed or predicted with high confidence. For those miRNAs (bta-miR-199c, miR-3431, miR-2299-5p, miR-152 and miR-1388-5p) without target information in IPA database, we used the perl scripts from the TargetScan website (http://targetscan.org) to predict (targetscan_60.pl) and calculate the context scores (targetscan_61_context_scores.pl) of their gene targets. Predicted targets with context + scores above 95^th^ percentile were further used. Predicted gene targets of DE miRNAs were then subjected to function and pathway analysis using IPA core analysis function. Targets with potential associations with lipid metabolism were used to produce gene networks.

### Quantitative PCR and data analysis

Quantitative RT-PCR was used to validate the expression of select differentially expressed miRNAs (bta-miR-199a-3p, miR-378, miR-34a and miR-98). Sequences of the most dominant isomiR of these miRNAs in our study were submitted to Exiqon (Exiqon Inc., USA) for use in designing the miRCURY LNA™ miRNA qPCR assays (product numbers 205968 [bta-miR-199a-3p], 204486 [bta-miR-34a], 205946 [bta-miR-378] and 204640 [bta-miR-98] used. Twelve samples (six/treatment) of day-14 and day + 28 were used for validation, as most of the miRNAs were significantly regulated at day + 28 when compared with day-14. Total RNA was reverse transcribed using Universal cDNA Synthesis Kit II (Exiqon). The cDNA was then quantified with the ExiLENT SYBR® Green Master Mix Kit using the miRCURY LNA™ assay according to manufacturer’s instructions. Bta-miR-103 (product No. 204063) was used as internal control as it was one of the most stable miRNA in our study (C.V = 11.4 %) and was also one of the reference assays suggested by Exiqon.

The expression of miRNA in a sample was normalized to bta-miR-103 and calculated relative to day-14 as follows: ΔCt_target miRNA_ = Ct_target miRNA_–Ct_bta-miR-103_; relative expression of target miRNA = 2-ΔΔCt, where ΔΔCt = ΔCt_target miRNA, day+28_ - ΔCt_target miRNA, day-14_. Significant differential expression was declared at *P* < 0.05. A repeated measures ANOVA model was used:

$$ {\mathrm{Y}}_{\mathrm{ijk}} = \upmu + {\mathrm{cow}}_{\left(\mathrm{i}\right)} + {\mathrm{time}}_{\mathrm{k}} + {\mathrm{e}}_{\mathrm{ik}} $$

where: μ = general mean; cow_(i)_ = random effect of cow j (experimental unit on which three measurements were made); time_k_ = time effect (k: −14, 28); e_ik_ = residual error.

## Results

### Milk fat percentage and individual fatty acid proportions during the period of linseed/safflower oil supplementation

**Fig. 1:**
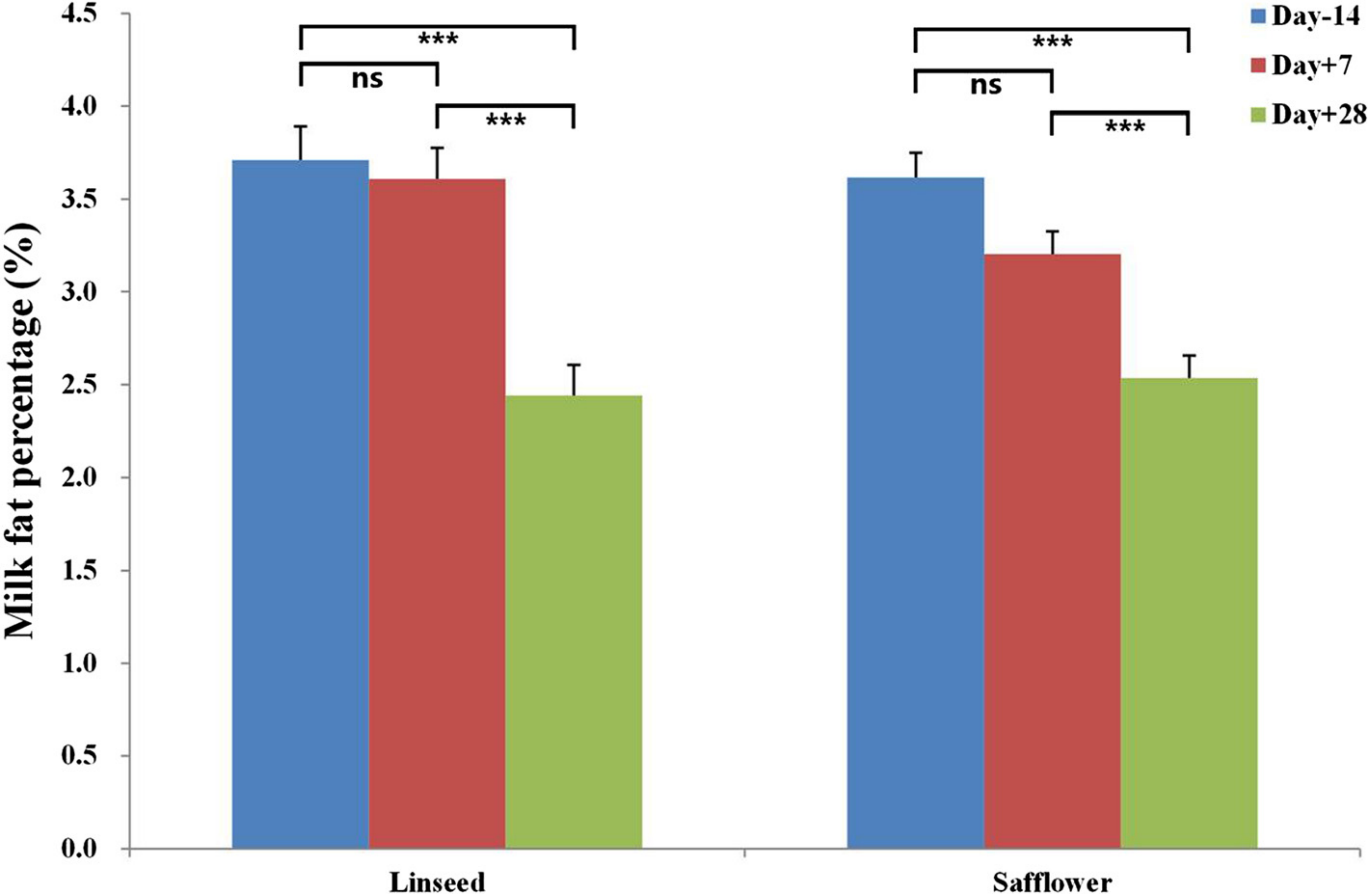
Response of test day milk fat percentage to 5 % linseed oil or 5 % safflower oil treatments (***P < 0.001; **P < 0.01, ns *P* > 0.05)

Dietary supplementation of cow diets with 5 % linseed oil reduced (*P* < 0.001) milk fat percentage from 3.711 % ± 0.183 in the control period (day-14) to 2.411 % ± 0.167 in the late treatment period (day + 28) while diets with 5 % safflower oil caused a reduction (*P* < 0.001) from 3.617 % ± 0.134 in the control period (day-14) to 2.536 % ± 0.121 in the late treatment period (day + 28) (Fig. 1). Five (C4:0, C6:0, C8:0, C14:0 and C16:0) out of seven saturated FAs measured and one USFA (C18:2n6tt) were significantly decreased by the two treatments (*P* < 0.05 to 0.0001) (Table 1). The concentration of C17:0 was also decreased by both treatments but significantly by linseed treatment only (*P* = 0.015) while C18:0 increased remarkable seven days after onset of treatment (significant for safflower treatment, *P* = 006) and dropped thereafter. The levels of five (C14:1, C18:1n11t, CLA:10t12c, C20:3n3 and C20:5n3) out of 13 USFAs measured increased significantly (*P* < 0.05 to ) in response to both treatments (Table 1). Significant (*P* < 0.05) increase in CLA:9c11t was recorded for linseed oil treatment only. The concentrations of C18:1n9c, C18:2n6cc and C18:3n3 were increased by both treatments but not significantly. The proportions of C14:1 t, C18:1n9t, C22:5n3 and C22:5n6 were unchanged by treatments.

**Table 1:** Change in milk fatty acid profiles (g/100 g) in response to 5 % linseed oil treatment or 5 % safflower oil treatment

	Linseed treatment^1^				Safflower treatment^1^			
FA	Day-14	Day + 7	Day + 28	*P*-value	Day-14	Day + 7	Day + 28	*P*-value
C4:0	2.476 ± 0.324^a^	0.787 ± 0.108^b^	0.986 ± 0.184^b^	<0.0001	2.276 ± 0.328^a^	1.261 ± 0.254^ab^	0.924 ± 0.105^b^	0.0034
C6:0	1.93 ± 0.260^a^	1.647 ± o.247^a^	0.753 ± 0.147^b^	0.0001	2.202 ± 0.171^a^	1.844 ± 0.165^a^	0.710 ± 0.178^b^	0.0000
C8:0	0.072 [0.048,0.106]^a^	0.038 [0.026, 0.057]^b^	0.022 [0.014, 0.035]^b^	<0.0001	0.077 [0.052, 0.112]^a^	0.041 [0.028, 0.060]^b^	0.029 [0.017, 0.051]^b^	0.0002
C14:0	13.161 ± 1.588^a^	8.004 ± 1.764^b^	5.726 ± 1.311^b^	0.0003	13.626 ± 2.035^a^	9.572 ± 1.596^ab^	6.283 ± 1.193^b^	<0.0001
C16:0	34.439 ± 6.052^a^	14.653 ± 4.656^ab^	8.502 ± 3.239^b^	<0.0001	31.419 ± 7.018^a^	13.141 ± 4.504^b^	15.662 ± 2.835^b^	0.0002
C17:0	2.809 ± 0.493^a^	1.643 ± 0.392^b^	1.157 ± 0.263^b^	0.0150	2.585 ± 0.660	1.792 ± 382	1.073 ± 0.234	0.0640
C18:0	4.080 ± 1.754	11.540 ± 2.741	3.491 ± 1.353	0.0804	5.430 ± 1.919^b^	15.916 ± 1.677^a^	5.923 ± 1.704^b^	0.0064
C14:1	1.198 [0.555, 2.608]^a^	0.266 [0.126, 0.561]^b^	0.107[0.049, 0.232]^c^	<0.0001	1.086 [0.527, 2.238]^a^	0.274 [0.133, 0.564]^b^	0.088 [0.041, 0.188]^b^	<0.0001
C14:1 t	0.022[0.015, 0.033]	0.014[0.009, 0.020]	0.010[0.007, 0.015]	0.145	0.024 [0.017, 0.033]	0.015 [0.011, 0.021]	0.013 [0.009, 0.018	0.1140
C18:1n9c	10.940 ± 3.066	13.174 ± 4.403	14.442 ± 3.614	0.4595	8.903 ± 2.167	21.429 ± 3.867	12.637 ± 2.861	0.1133
C18:1n9t	0.191 ± 0.025	0.216 ± 0.024	0.147 ± 0.024	0.8863	0.121 ± 0.033	0.200 ± 0.032	0.137 ± 0.020	0.4986
C18:1n11t	0.023 [0.008, 0.066]^b^	0.160[0.055, 0.462]^a^	0.175 [0.061, 0.505]^a^	<0.0001	0.028 [0.010, 0.078]^b^	0.087 [0.031, 0.241]^ab^	0.368 [0.128, 1.509]^a^	0.0003
CLA:9c11t	0.047 [0.032, 0.068]^b^	0.053 [0.036, 0.076]^ab^	0.069 [0.047, 0.100]^a^	0.0347	0.045 [0.022, 0.090]	0.054 [0.027, 0.109]	0.072 [0.035, 0.149]	0.2735
CLA:10t12c	0.014 [0.008, 0.023]^b^	0.071 [0.042, 0.120]^a^	0.047 [0.028, 0.080]^a^	<0.0001	0.020 [0.014, 0.029]^b^	0.034 [0.024, 0.049]^a^	0.038 [0.026, 0.0562]^a^	0.0106
C18:2n6cc	0. 064 [0.025, 0.165]	0.186 [0.072, 0.481]	0.051 [0.020, 0.132]	0.1430	0.101 [0.034, 0.303]	0.281 [0.094, 0.840]	0.071 [0.023, 0.223]	0.1080
C18:2n6tt	0.173 [0.062, 0.328]^a^	0.018 [0.010, 0.032]^b^	0.030 [0.016, 0.055]^b^	0.0000	0.219 [0.138, 0.349]^a^	0.083 [0.052, 0.132]^a^	0.028 [0.017, 0.046]^b^	0.0000
C18:3n3	0.068 [0.028, 0.164]	0.164 [0.068, 0.395]	0.249 [0.103, 0.600]	0.0950	0.070 [0.030, 0.162]	0.115 [0.050, 0.266]	0.180 [0.075, 0.431]	0.5620
C20:3n3	0.004 [0.002, 0.008]^b^	0.014 [0.008, 0.025]^ab^	0.022 [0.012, 0.041]^a^	<0.0001	0.004 [0.002, 0.007]^b^	0.006 [0.003, 0.010]^ab^	0.019 [0.011, 0.034]^a^	0.0004
C20:5n3	0.007 [0.005, 0.011]^b^	0.017 [0.012, 0.026]^ab^	0.019 [0.013, 0.029]^a^	<0.0001	0.007 [0.004, 0.010]^b^	0.006 [0.004, 0.009]^b^	0.013 [0.008, 0.020]^a^	0.043
C22:5n3	0.005 [0.002, 0.009]	0.009 [0.005, 0.018]	0.014 [0.007, 0.027]	0.0760	0.007 [0.003, 0.014]	0.005 [0.003, 0.011]	0.007 [0.003, 0.015]	0.2154
C22:5n6	0.020 [0.012, 0.033]	0.011 [0.007, 0.018]	0.016 [0.010, 0.027]	0.6767	0.010 [0.007, 0.017]	0.015 [0.010, 0.024]	0.013 [0.008, 0.022]	0.7957

Note: ^1^The mean values of C4:0, C6:0, C14:0, C16:0, C17:0, C18:0, C18:1n9c, C18:1n9t are represented as MEAN ± SEM, while those of C8:0, C14:1, C14:1 t, C18:1n11t, C18:2n6cc, C18:2n6tt, C18:3n3, C20:3n3, C20:5n3, C22:5n3, C22:5n6, CLA:t10c12 and CLA:c9t11 are represented as MEAN (which is back transformed from the mean of logged mean value) with 95 % confidential intervals. ^a-c^For each treatment, means within a row with different superscripts differ significantly (*P* < 0.05)

### High-throughput sequencing of small RNA libraries prepared from bovine mammary gland tissues

**Fig. 2:**
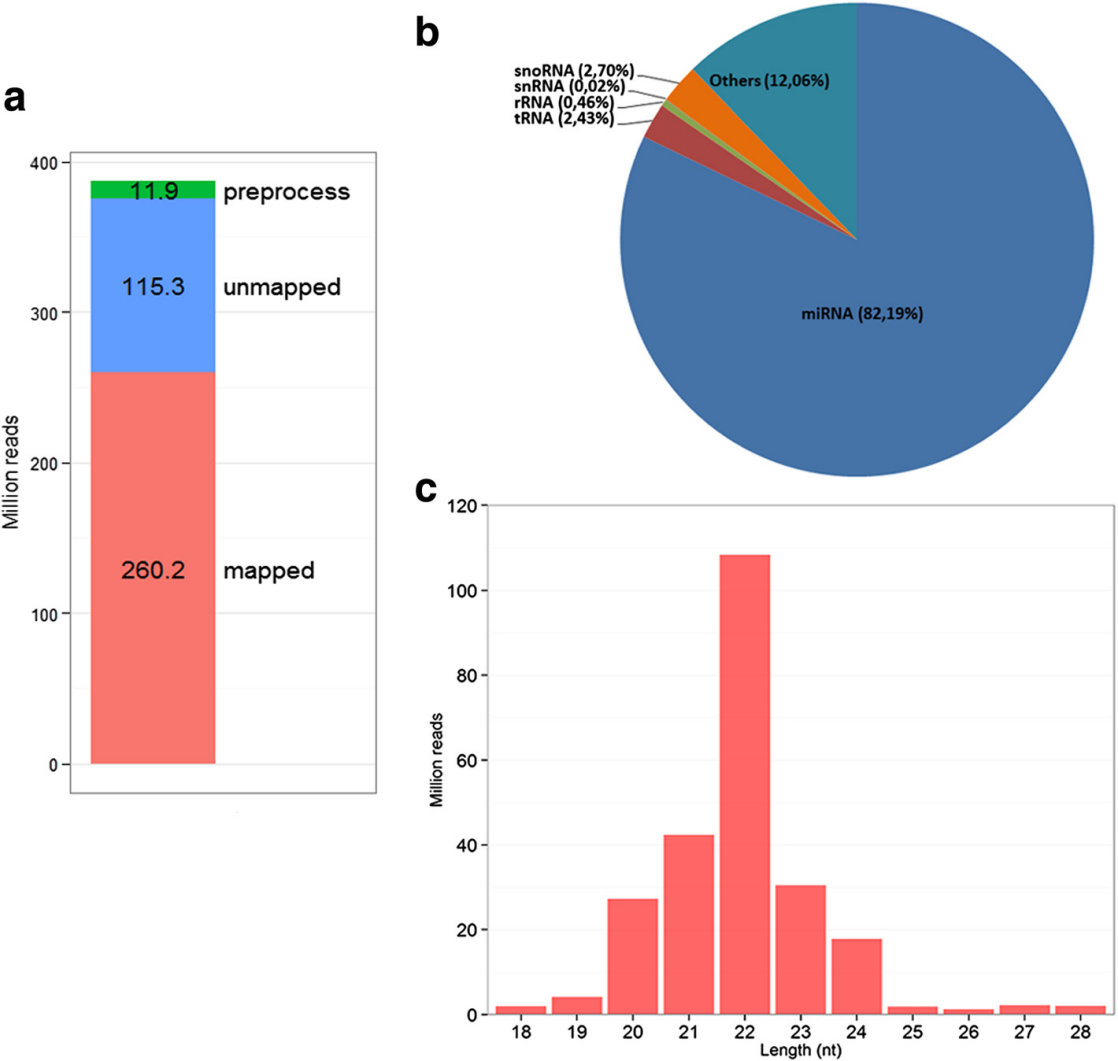
Raw data filtering and mapping statistics. **a** The statistics of raw reads from the preprocessing and mapping steps. A total of 387.4 million raw reads were processed. A total of 11.9 million reads were filtered during the preprocessing step (*aqua bar*). Reads that could not map to the bovine genome were 115.3 million (*purple bar*). This number (115.3 million) includes reads which mapped to more than 5 genomic locations. Finally, reads that mapped successfully to the bovine genome (Bta_4.6.1) were 260.2 million (*red bar*). **b** The relative abundance of different classes of small RNAs in the total reads that were successfully mapped to the bovine genome; (**c**) Length distribution of the mapped reads across all the libraries

Thirty five libraries out of 36 were successfully sequenced, yielding more than 387 million raw reads with an average of 11 million reads per sample (Fig. 2a and Additional file 3). One library (cow 62 at day + 28) with only 15,149 raw reads was discarded and not further processed. Following adaptor removal, quality filtering and removal of short sequences (<18 nt), more than 361 million reads (361,597,513 reads) were retained for further analysis. Out of this number, more than 260 million reads (about 67.2 % of the total reads) were successfully mapped to the bovine genome (Btau_4.6.1) (Fig. 2a). A small proportion of the mapped reads (5.6 %) were further classified into different small RNA categories (rRNA, tRNA, snRNA and snoRNA) (Fig. 2b). The remaining reads (245,563,876) were used to profile known miRNAs and to discover novel miRNAs using miRDeep2 [36]. The length distribution of the mapped reads showed a sharp peak at 22 nt (Fig. 2c).

### Known miRNA identification and genomic expression pattern

**Fig. 3:**
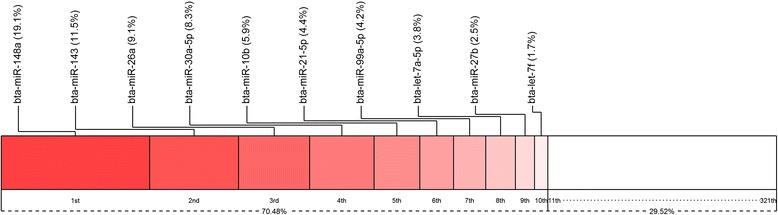
Top10 highly expressed miRNAs in bovine mammary gland following supplemental feeding with 5 % linseed oil and 5 % safflower oil

**Fig. 4:**
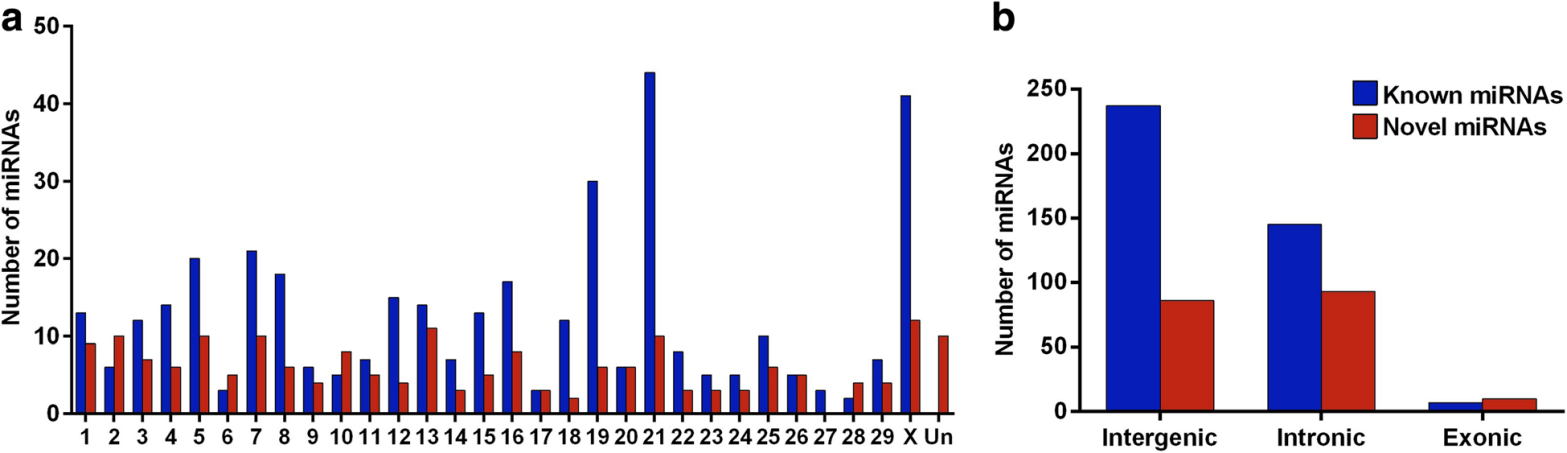
Distribution of known miRNAs (*blue bar*) and novel miRNAs (*red bar*) across bovine chromosomes (**a**) and bovine genomic regions (**b**)

Based on miRBase Release 21, we identified a total of 321 miRNAs with an abundance of more than one CPM in at least ten libraries (Additional file 4). MiRNAs were the predominant small RNA species, accounting for 82.2 % of mapped reads (Fig. 2b). Ten miRNAs were highly expressed, accounting for about 70.5 % of all the known miRNAs (Fig. 3) with similar expression levels across all the libraries (Additional file 5), while the remaining miRNAs were expressed at much lower levels. The genomic distribution of the detected miRNAs was diverse among all the chromosomes (Chr). A large number of miRNAs were located on Chr 21 (44 miRNAs), X (41 miRNAs), and 19 (30 miRNAs), while only 11 miRNAs in total were mapped on Chr 6, 17, 27 and 28 (Fig. 4a). Most miRNAs (195) were located within intergenic regions, followed by intronic regions (118) while only a small number (7) were found within exons (Fig. 4b).

Furthermore, we compared the dominant isomiRs of each miRNA in our study with those in miRBase (Release 21). Our results indicate that, the dominant isomiRs of more than half the miRNAs in our study (194 out of 321) differed from the consensus sequences deposited in miRBase (Release 21) (Additional file 6), while only 127 (out of 321) miRNAs share the same consensus sequences as in miRBase (Release 21). To understand the reason for this discrepancy, we conducted global modification analysis [37] of the dominant isomiR sequences in our study and found out that single nucleotide 3’extensions were the most prevalent type of modifications while 5’end modifications were much lower (Additional file 7a). The prevalent 3’extensions were U and A nucleotides. Internal modification analysis showed that deamination of adenosine to inosine bases at positions 4 to 20 by adenosine deaminases was the only type of internal modification present (Additional file 7b). We also examined the arm selection of the precursors of miRNAs in our study using the formula *ω* = 5p/(5p + 3p) as a measure for arm selection [41]. Out of the 293 precursors encoding the 321 miRNAs identified in this study, 114 were predominantly expressed at the 5’ arm (0.9 ≤ *ω* ≤ 1) and 107 at the 3’ arm (0 ≤ *ω* ≤ 0.1) as well as 72 precursors co-expressed at both arms (0.1 < *ω* < 0.9) (Additional file 8).

### Novel miRNA discovery

In this study, miRDeep2 score of 5 which produced a signal-to-noise ratio of 15.7 was used as the cut-off value for novel miRNA prediction [42] (Additional file 9a). Using this cut-off value and miRBase (Release 21), 261 novel miRNAs were initially detected by miRDeep2. In order to identify novel miRNAs with high confidence, only those novel miRNAs with more than 10 total counts and present in at least three libraries were considered true novel miRNAs. Based on our criteria, 187 novel miRNA precursors coding for 176 putatively novel mature miRNAs (Additional file 9b) were identified in this study. Novel miRNAs that did not fit our criteria were considered miRNA candidates (Additional file 9c). The highest number of novel miRNAs were found on Chr X (12 miRNAs), followed by Chr 13 (11 miRNAs) with the least number on Chr 8 (6 miRNAs) (Fig. 4a). Chr 27 was the only chromosome devoid of novel miRNAs. Furthermore, we also identified five novel and two already reported [43] miRNA precursors that were reversely complementary to known bovine miRNA precursors (Additional file 9d). Additionally, two novel mature miRNAs, miR-nv163 and miR-nv185, were reversely complementary to known miRNAs (bta-miR-194 and miR-338, respectively).

### Differentially expressed miRNAs in response to linseed oil supplementation

The correlations between libraries (day-14, day + 7 and day+28) of each cow were calculated using the normalized counts of expressed miRNAs. The lowest correlation between different time points of each cow in linseed oil treatment was R^2^ = 0.956, indicating a very high correlation between all the libraries (Additional file 10a). The expression of 14 miRNAs were significantly altered (*P* < 0.05) at day + 28 (treatment period) as compared to the control period (day-14) (Table 2). Out of this number, 11 were up-regulated (bta-miR-4286, miR-885, miR-199c, miR-199a-3p, miR-3431, miR-98, miR-196a, miR-378, miR-23b-3p, miR-148b and miR-21-5p) while only 3 were down-regulated (miR-200a, miR-335 and miR-2299-5p) (Table 2). All the significantly DE miRNAs demonstrated a subtle change ranging from 1.28 to 1.73 fold. No significantly DE miRNAs were identified at day+7 (one week after onset of treatment) as compared to Day-14.

**Table 2:** Differentially expressed miRNAs in response to 5 % linseed oil or 5 % safflower oil treatments

miRNA	Linseed treatment					Safflower treatment				
	Log2FC^1^	FC^2^	logCPM^3^	*P*-value	FDR^4^	Log2FC	FC	logCPM	*P*-value	FDR
**bta-miR-148b**	0.365	1.288	12.16	0.001	0.028	0.604	1.520	12.31	0.000	0.001
**bta-miR-199a-3p**	0.558	1.472	12.05	0.000	0.000	0.857	1.811	12.11	0.000	0.000
**bta-miR-199c**	0.589	1.504	7.93	0.000	0.000	0.984	1.977	7.99	0.000	0.000
**bta-miR-21-5p**	0.352	1.277	15.46	0.001	0.031	0.653	1.572	15.36	0.000	0.000
**bta-miR-378**	0.414	1.332	8.76	0.001	0.031	0.736	1.666	8.85	0.000	0.000
**bta-miR-98**	0.505	1.419	8.98	0.000	0.002	0.796	1.736	8.95	0.000	0.000
bta-miR-152	-	-	-	-	-	0.551	1.465	10.62	0.000	0.002
bta-miR-16a	-	-	-	-	-	0.529	1.443	8.60	0.001	0.011
bta-miR-28	-	-	-	-	-	0.525	1.439	9.59	0.000	0.004
bta-miR-34a	-	-	-	-	-	0.940	1.919	5.87	0.001	0.011
bta-miR-145	-	-	-	-	-	−0.473	−1.388	13.29	0.002	0.029
bta-miR-99a-5p	-	-	-	-	-	−0.487	−1.402	15.32	0.000	0.004
bta-miR-196a	0.444	1.361	6.07	0.002	0.050	-	-	-	-	-
bta-miR-23b-3p	0.401	1.320	10.77	0.001	0.031	-	-	-	-	-
bta-miR-3431	0.532	1.446	6.36	0.002	0.049	-	-	-	-	-
bta-miR-4286	0.787	1.725	4.18	0.000	0.000	-	-	-	-	-
bta-miR-885	0.782	1.719	4.80	0.001	0.035	-	-	-	-	-
**bta-miR-200a**	−0.470	−1.385	13.85	0.000	0.001	−0.521	−1.435	13.99	0.000	0.000
bta-miR-335	−0.581	−1.496	6.95	0.000	0.020	-	-	-	-	-
bta-miR-2299-5p	−0.890	−1.853	3.76	0.001	0.028	-	-	-	-	-
bta-miR-125b	-	-	-	-	-	−0.563	−1.477	13.09	0.001	0.016
bta-miR-99b	-	-	-	-	-	−0.573	−1.488	11.43	0.001	0.012
bta-miR-125a	-	-	-	-	-	−0.574	−1.489	12.22	0.002	0.032
bta-miR-96	-	-	-	-	-	−0.606	−1.522	11.60	0.000	0.000
bta-miR-484	-	-	-	-	-	−0.709	−1.635	7.79	0.000	0.005
bta-miR-1388-5p	-	-	-	-	-	−0.749	−1.681	6.81	0.000	0.006
bta-miR-342	-	-	-	-	-	−0.830	−1.778	8.75	0.000	0.004
bta-miR-486^3^	-	-	-	-	-	−1.754	−3.373	7.94	0.000	0.000
bta-miR-1271	-	-	-	-	-	−2.262	−4.797	6.61	0.000	0.009

^1^Log2FC: log2 fold change; ^2^FC: fold change; ^3^logCPM: log2-counts per million; ^4^Benjaminin Hochberg FDR p-values.

The miRNAs in bold are among core differentially expressed miRNAs which were found in both treatments.

bta-miR-486 was also found to be differentially expressed at day + 7 as compared to day-14 in safflower treatment

### Differentially expressed miRNAs in response to safflower oil supplementation

For cows supplemented with safflower oil, there was also a high correlation (R^2^ ≥ 0.964) between libraries (Additional file 10b). When compared with the control period (day-14), we identified a total of 22 DE miRNAs at day+28 including 10 up-regulated (bta-miR-199c, miR-199a-3p, miR-98, miR-378, miR-21-5p, miR-148b, miR-34a, miR-152, miR-16a, and miR-28) and 12 down-regulated (bta-miR-200a, miR-145, miR-99a-5p, miR-125b, miR-99b, miR-125a, miR-96, miR-484, miR-1388-5p, miR-342, miR-486 and miR-1271) (Table 2). Among the DE miRNAs, bta-miR-1271 was the most affected showing a 2.26 fold down-regulation followed by bta-miR-486 with a 1.75 fold decrease. Except for bta-miR-1271 and miR-486, the majority of the DE miRNAs showed subtle changes in fold gene expression. Additionally, only the expression of bta-miR-486 was significantly affected at day+7 (7 days following onset of treatment) as compared to the control period (day-14), showing a 2.84 fold down-regulation. Seven of the DE miRNAs by safflower oil treatment (6 up-regulated: bta-miR-199c, miR-199a-3p, miR-98, miR-378, miR-148b, miR-21-5p; one down-regulated: bta-miR-200a) were also significantly affected by linseed oil supplementation. However, the effect of safflower oil treatment on miRNA expression in this study was more pronounced (22 DE miRNAs) as compared to linseed oil treatment (14 DE miRNAs) (Table 2).

### Real-time quantitative PCR validation of identified miRNA expression profiles

**Fig. 5:**
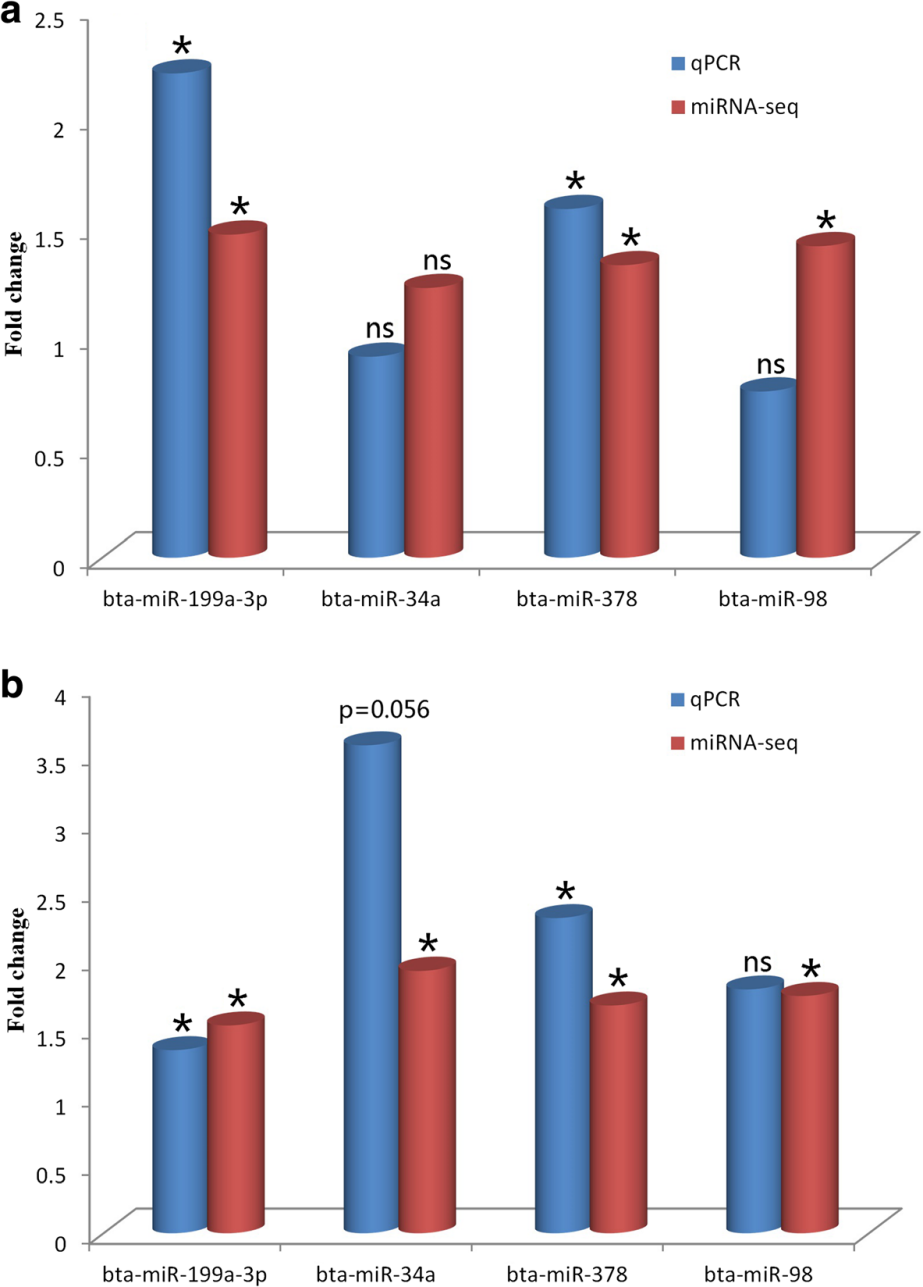
Results of qPCR validation of the expression of four miRNAs in linseed (**a**) and safflower (**b**) treatments, when comparing day+28 with day-14

Real-time quantitative PCR (qPCR) was used to validate the expression levels of selected DE miRNAs (bta-miR-199a-3p, miR-34a, miR-378 and miR-98) identified in this study (Fig. 5). Expression trends of selected miRNAs were generally similar to the results from miRNA-sequencing. For example, bta-miR-199a and bta-miR-378 were differentially expressed at day+28 compared with day-14 in both treatments (*P* < 0.05) after RNA-sequencing and confirmed by qPCR. Bta-miR-34a in linseed treatment was not significantly differentially expressed using qPCR and miRNA-sequencing. The expression level of bta-miR-34a was higher with qPCR detection but only approached significance (*P* = 0.056) as compared to RNA-sequencing results. The only exception was the qPCR results of bta-miR-98, which showed a reversed trend from RNA-sequencing results in linseed treatment.

### Target gene prediction of differentially expressed miRNAs and functional annotation

In order to explore possible roles of the DE miRNAs in mammary lipogenesis, we used Ingenuity Pathway Analysis (IPA) software to predict DE miRNA gene targets and to investigate the enriched functions which might be impacted by the alterations of miRNA expression by diets rich in USFAs. Since there is a high false positive rate for miRNA target prediction, we only analyzed the targets with high confidence (highly predicted or experimentally observed).

Our analysis indicates that, about 3594 genes could be targeted by the eleven up-regulated miRNAs (bta-199a-3p, miR-98, miR-378, miR-21-5p, miR-148b, miR-4286, miR-885, miR-196a, miR-23b-3p, bta-miR-199c and miR-3431) whereas 1163 genes could be targeted by the three down-regulated miRNAs (bta-miR-335, miR-200a and bta-miR-2299-5p) in linseed oil-treated cows. Predicted gene targets were further subjected to core analysis to explore their functions. The targets of up-regulated and down-regulated miRNAs were associated (*P* < 0.05) with 18 and 16 molecular/cellular functions respectively (Additional file 11). The most significantly enriched functions for all the gene targets for both up-regulated and down-regulated DE miRNAs were related to gene expression, followed by functions associated with general cellular metabolism such as cellular growth and proliferation, cellular development, and cellular morphology.

In the safflower oil-treated group, the ten up-regulated (bta-miR-199a-3p, miR-98, miR-378, miR-21-5p, miR-148b, miR-34a, miR-16a, miR-28, miR-199c and miR-152) and nine down-regulated (bta-miR-200a, miR-145, miR-99b, miR-125a, miR-484, miR-342, miR-486, miR-1271 and miR-1388-5p) miRNAs could target respectively 1610 and 1300 genes with high confidence while three miRNAs (bta-miR-99a-5p, miR-125b and miR-96) only have targets with low confidence in IPA knowledge base and were not considered for further analysis. Gene targets for both up- and down-regulated miRNAs were most enriched in functions related to gene expression (*P* < 0.05), followed by general cellular metabolism functions similar with those of linseed treatment (Additional file 11).

### Functional annotation of core differentially expressed miRNAs shared by the two treatments

**Fig. 6:**
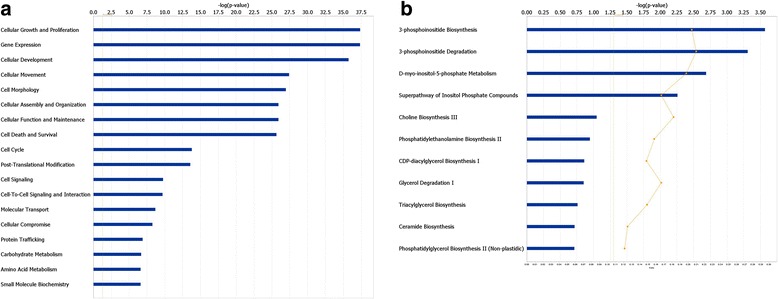
Functional annotation and enriched canonical pathways of core differentially expressed miRNA targets. **a** Functional annotation of all the targets by core differentially expressed miRNAs (**b**) Enriched canonical pathways related with lipid metabolism

**Fig. 7:**
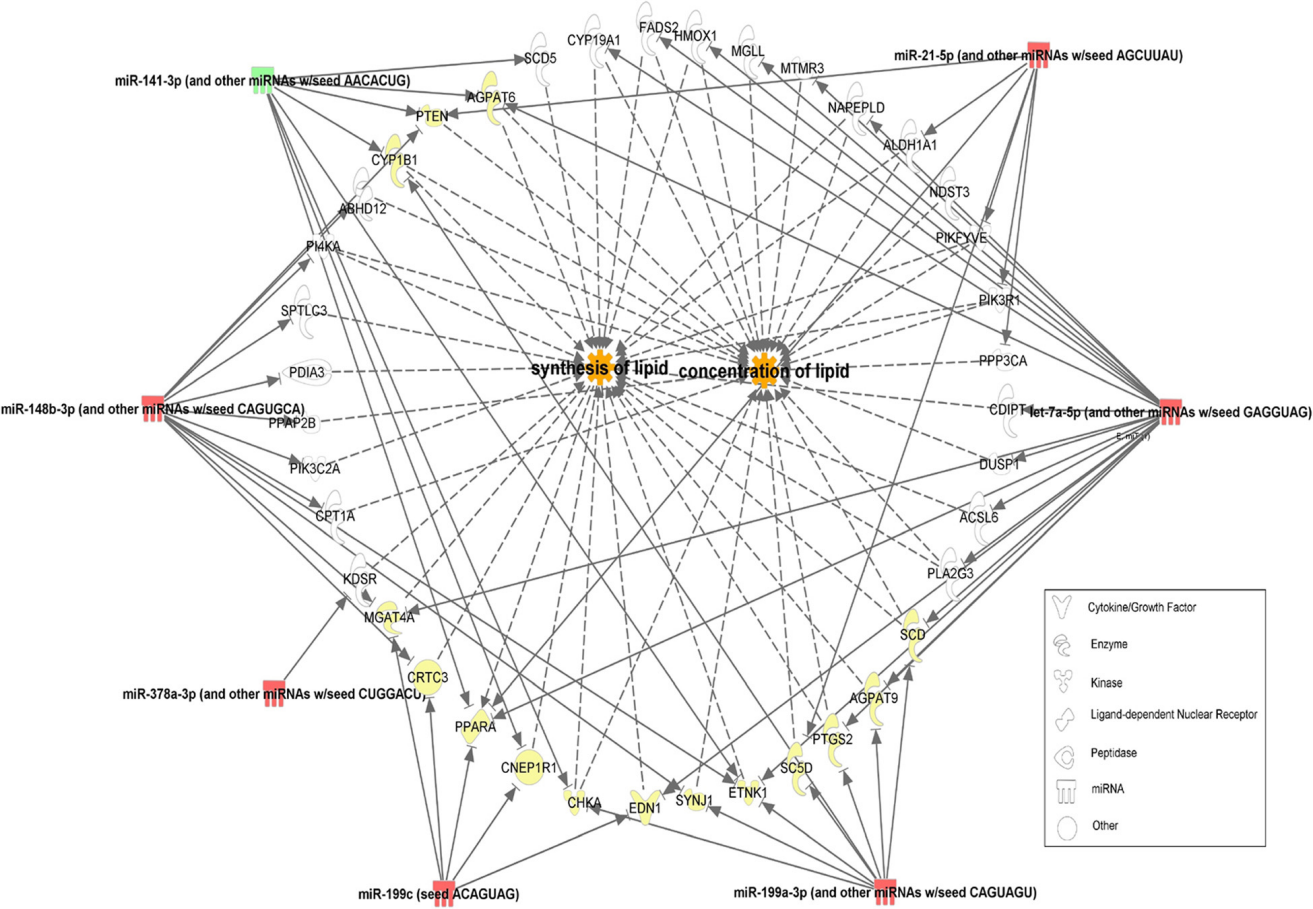
Two functions of core miRNAs with the highest number of targets related with lipid metabolism. Red color indicates up-regulated miRNAs; Green color indicates down-regulated miRNAs; Yellow color shows genes targeted by more than one miRNAs

The expression of seven miRNAs including six up-regulated (bta-miR-199c, miR-199a-3p, miR-98, miR-378, miR-148b and miR-21-5p) and one down-regulated (bta-miR-200a) were significantly affected by both treatments. Since both treatments significantly induced milk fat depression, the common DE miRNAs identified in this study were more likely to have critical roles in the regulation of mammary lipogenesis and thus considered core DE miRNAs. The seven core DE miRNAs could target 2922 genes with high confidence. The highest number of gene targets (1180) was recorded for bta-miR-98 while miR-199a-3p only targeted 81 genes (with high confidence). The most significantly enriched function by target genes of core DE miRNAs was cellular growth and proliferation, followed by gene expression (Fig. 6a). We further explored the pathways associated with lipid metabolism. Among all the affected canonical pathways, four including 3-phosphoinositide biosynthesis, 3-phosphoinositide degradation, D-myo-inisitol-5-phosphate metabolism and the superpathway of inositol phosphate compounds, were related with lipid metabolism (*P* < 0.05) (Fig. 6b). The IPA filter function enabled us to further identify the target genes that are associated with lipid metabolism. The largest number of target genes (39) were associated with two functions (synthesis of lipid and concentration of lipid) related with lipogenesis (Fig. 7).

## Discussion

In this study, we examined the effect of linseed/safflower oil treatments on the miRNome expression in bovine mammary glands using high throughput miRNA sequencing method. Feeding ingredients with high concentrations of USFAs (linseed oil, soybean oil, corn oil, safflower oil, etc.) have been considered as an efficient strategy to alter the milk FA profiles of bovine milk in favor of health promoting components, mainly the increase in USFAs including CLA, α-linolenic acid and linoleic acid [5, 8–10, 44]. These reports are supported by our findings. The increased beneficial USFA contents recorded in this study could be associated with the altered expression of mammary lipogenic enzymes [45, 46]. The concentration of trans-FA and CLA isomers in milk are negatively correlated with the abundance of key lipogenic genes and transcription factors such as peroxisome proliferator-activated receptor gamma (PPARγ) and sterol regulatory element binding factor 1 (SREBP1) [47]. MiRNAs are universal regulators of gene expression at post-translational level and thus are potentially involved in the mammary adaptation process to increased availability of dietary USFAs and modulation of the pathways of lipogenesis. Deep sequencing of six samples per treatment produced an average of more than 10 million reads per sample, enabling us to profile the miRNA expression with high depth and identify miRNAs significantly affected by the diets with high confidence.

We identified 321 miRNAs with an average count greater than one CPM in at least ten libraries, accounting for 40.35 % of all known bovine miRNAs deposited in miRBase (Release 21). Six (bta-miR-148a, miR-26a, miR-21-5p, miR-27b, le-7f and let-7a-5p), four (bta-miR-30a-5p, miR-26a, miR-21-5p and let-7a-5p) and five (bta-miR-148a, miR-26a, let-7a-5p, miR-143 and miR-21-5p) of the highly expressed miRNAs in our study are also among the top 10 highly expressed miRNAs detected respectively in bovine mammary epithelial cells (MAC-T) [43] and lactating glands [24, 48]. Our results and these reports suggest that these highly abundant miRNAs may be implicated in the lactation process, milk synthesis and in mammary gland biology. Previous studies have already unveiled the roles of some of these highly expressed miRNAs in mammary glands. For example, miR-148a and miR-143 are highly expressed in both bovine and goat mammary glands during lactation [49]; miR-148a and miR-26a have been shown to demonstrate consistent expression patterns in bovine milk throughout the lactation period [50] and miR-21-5p increased in expression remarkably at fresh period compared with dry period [23]. Accumulating evidence support the notion that miR-148a can promote cell proliferation and differentiation [51, 52]. Another highly expressed miRNA (miR-21-5p) displayed a marked increase in expression during early lactation indicating that it might be involved in promoting mammary cell proliferation during early lactation [23]. Furthermore, miR-148a can repress WNT (Wingless/INT-1) signaling and thus promote adipogenesis [53] while miR-27b can repress human adipocyte differentiation by directly targeting PPARγ [54]. Repression of miR-143 by its antisense sequence inhibited differentiation of preadipocytes by down regulating ERK5 (also known as MAPK7-mitogen-activated protein kinase 7), indicating a role for this miRNA in adipocyte differentiation [55].

A total of 187 novel miRNA precursors encoding 176 novel miRNAs were detected with high confidence in this study as well as 46 novel miRNAs candidates (had miRDeep2 score higher than 5 but present in less than 3 libraries). Novel miRNAs discovered by high throughput sequencing data have become the main contributors to the miRNome repertoire since 2008 [56]. Considering the number of bovine miRNAs (793 miRNAs) in the miRBase (Release 21), our results will greatly increase the repertoire of bovine miRNAs. Besides, we also found that eight novel precursors were reversely complementary to known bovine miRNA precursors and two novel mature miRNAs were reversely complementary to the known mature miRNAs, which confirmed previous results [43]. This kind of complementation cannot be clearly represented by the current miRNA naming scheme [56–58] which needs to be improved. In this study, we found out that U and A modifications at the 3’end of sequences are the dominant variations, and could explain the differences between the sequences of the dominant isomiRs of 60 % (194 out of 321 miRNAs) of the miRNAs in our study as compared to their miRBase consensus sequences. Previous studies have reported that post-transcriptional alterations modify the 3’ends of miRNAs via mono/poly-uridylation, polydenylation, adenylation as well as other forms of modifications [59–62]. It is also thought that such modifications may modulate miRNA targeting effectiveness [59]. Taken together, the modifications observed in our data may have functional significance which however needs further investigation.

We have investigated the response of miRNA expression of bovine mammary glands to diets supplemented with linseed oil and safflower oil. Linseed oil is rich in α-linolenic acid (57 % of total fat) while safflower oil is rich in linoleic acid (76 % of total fat). Nevertheless, dietary supplementation with the two oil types introduced similar levels of milk fat reductions of ~30 % which is in concordance with previous studies that saw reductions of up to 50 % in milk fat yield [6, 9, 63]. When compared with the control period, only one miRNA was significantly affected at day+7 with safflower supplementation while no miRNAs were significantly affected at day+7 with linseed supplementation. This might be the result of subtle changes at day+7 that could not be detected. Results on milk fat production showed no significant reductions in test day milk fat percentage seven days after onset of treatment with linseed and safflower oils. Subsequently, significant reductions in test day fat percentage were evident 28 days after onset of treatment which coincided with the time when the most miRNAs were significantly differentially regulated by treatments. The seven core DE miRNAs were common to both treatments, making them more likely to be involved in the regulation of lipogenesis in response to USFAs. Functional annotation of their targets showed that these miRNAs were highly enriched in functions associated with cellular metabolism as well as gene expression. Furthermore, four pathways relating to lipid metabolism were also significantly enriched (*P* < 0.05), implying that these miRNAs could be critical players in these pathways. It should be noted that some of the core DE miRNAs in this study (miR-98, miR-21-5p and miR-200a) have been previously suggested to play important roles in the adipogenesis network [64, 65]. miR-21-5p can negatively regulate PPARα, which is a transcription factor and a major regulator of genes encoding lipid metabolizing enzymes [66]. Elevated expression of miR-21-5p can repress the lipid metabolism pathway regulated by PPARα [67]. Furthermore, miR-200a/miR-8 cluster has been shown to negatively regulate WNT signaling which blocks adipogenic differentiation in multipotent mesenchymal stem cells [68]. Moreover, some important lipogenic enzymes were predicted to be potential targets of the core DE miRNAs in this study. Target analysis showed that stearoyl-CoA desaturases, SCD1 and SCD5, which are involved in FA biosynthesis, are targeted by bta-miR-200a and miR-199a-3p respectively. FADS2, which cause desaturation of FAs is a direct target of bta-miR-98a (IPA knowledge base) while miR-378 can regulate adipocyte differentiation by directly targeting PPARγ and C/EBPα (CCAAT/enhancer-binding protein α), which promote lipogenesis stimulation and increase lipid droplet size in developing adipocytes when overexpressed [69]. Interestingly, six of the seven core DE miRNAs were up-regulated, from which we could expect the repression of a large number of mRNAs related with FA synthesis. This expectation agrees with a previous study which found that feeding conjugated linoleic acid (CLA) repressed milk fat synthesis accompanied by inhibition of the expression of many genes involved in milk lipid synthesis [70]. SCD1 gene, presumably targeted by bta-miR-199a-3p is a key gene with role in the synthesis of USFAs, was reported to be down-regulated in response to linseed oil supplementation [18]. Although we constrained the targets of core DE miRNAs to a small number in this study and demonstrated that they might regulate FA synthesis by targeting lipogenic genes, the involvement of most of the miRNAs in bovine mammary lipogenesis still need to be further validated.

In this study, safflower oil treatment was accompanied by more DE miRNAs (22 DE miRNAs) than linseed oil treatment (14 DE miRNAs) suggesting that there might be treatment-specific effects on the expression of miRNAs, which can further influence expression of lipogenic genes and pathways. Dietary lipids entering the rumen are first transformed to free FAs by microbial lipases, which produce the intermediates for biohydrogenation [71]. Due to the distinctions in chemical structure, the transformation of α-linolenic acid (3 double bonds in their structure) and linoleic acid (2 double bonds) during the biohydrogenation process may require different metabolic pathways [72]. Despite the fact that their biohydrogenation pathways are not fully clarified, it is well acknowledged that they produce diverse intermediates of biohydrogenation [73], which may affect the mammary milk fat synthesis pathway differently. These treatment-specific effects could explain the differential number of significantly regulated miRNAs by the two treatments (Table 2). The functions of most of the DE miRNAs specific to each treatment remain largely unknown. miR-335 is up-regulated in response to lipid loading and is highly abundant in liver and adipose tissues of obese mice, suggesting a role in lipid metabolism [74]. miR-196 has a higher expression in visceral than subcutaneous fat in beef cattle, showing tissue specificity, which could be associated with adipogenesis [29]. miR-342 can inhibit the expression of SREBP, resulting in down-regulation of FASN and 3-hydroxy-3-methylglutaryl CoA reductase (HMGCR) and inhibition of FA biosynthesis in prostate cancer cells [75]. It should also be noted that two of the DE miRNAs in linseed group (bta-miR-2299-5p and miR-3431) are ruminant specific [76, 77]. Bta-miR-2299-5p is one of bovine specific miRNAs associated with insulin signaling pathway [76] whereas involvement of bta-miR-3431 in postnatal muscle development has been demonstrated [77]. These bovine specific miRNAs shape ruminant specific phenotypes [76], indicating that they might also play roles in ruminant specific lipid synthesis pathways, which however need further verification.

## Conclusion

In this study, dietary supplementation with 5 % linseed oil or 5 % safflower oil induced significant milk fat reductions, decreased significantly the proportions of some individual SFAs while the proportions of some individual USFAs were significantly increased. Twenty-two miRNAs were significantly differentially expressed in response to the treatments, indicating that miRNAs could be important regulators of bovine mammary lipogenesis. Core differentially regulated miRNAs by both treatments suggest roles in regulating lipogenic pathways or mammary gland functions. The abundant novel miRNAs we identified will further enrich the bovine miRNome repertoire and contribute in the understanding of mammary gland biology.

## Additional files

Additional file 1:
**Composition of the experimental diets.** (DOCX 16 kb) Additional file 2:
**Library barcodes of miRNA-seq library preparation.** (DOCX 16 kb) Additional file 3:
**Mapping statistics of reads by library.** (XLSX 16 kb) Additional file 4:
**Known miRNAs and number of reads per library per sampling point.** (XLSX 78 kb) Additional file 5:
**Top expressed miRNAs at different time points according to treatments.** (DOCX 17 kb) Additional file 6:
**Summary of most abundant isomiRs of detected miRNAs.** (XLSX 36 kb) Additional file 7:
**Summary of modification analysis of dominant isomiRs that differed from consensus miRBase sequences.** (A) 5’end (5p) and 3’ end (3p) modifications of isomiRs showed that single nucleotide 3’extensions are dominated by U and A modifications while 5’end modifications are much lower. (B) Internal modifications are due to deamination of adenosine to inosine by adenosine deaminases (ADAR). The start of a miRNA on the x-axis is at index ‘0’ (5' end) while its end is at index ‘22’ (3' end). Note: U, A, C and G refer to additions; G_adar refers to deamination of an adenosine to inosine by adenosine deaminases ; U_snp, A_snp, C_Snp and G_snp refer to snps which are different from the genome. (PNG 212 kb) Additional file 8:
**Summary of dominant arm selection of detected miRNAs.** (XLSX 34 kb) Additional file 9:
**mirDeep2 results of known miRNA identification and novel miRNA discovery; (** **A** **) miRDeep2 performance for score cut-offs −10 to 10, (** **B** **) novel miRNA information, (** **C** **) candidate novel miRNAs and (** **D** **) novel Precursors which were reversely complementary to known precursors.** (XLSX 76 kb) Additional file 10:
**Pearson correlation of the libraries in (** **A** **) safflower and (** **B** **) linseed oil treatments.** (XLSX 58 kb) Additional file 11:
**Functional annotation of the targets of (** **A** **) up-regulated and (** **B** **) down regulated miRNAs in linseed oil treatment, and (** **C** **) up-regulated and (** **D** **) down-regulated miRNAs in safflower oil treatment.** (PDF 290 kb)
