# Supplementary material for: Deep sequencing shows microRNA involvement in bovine mammary gland adaptation to diets supplemented with linseed oil or safflower oil
Source: BMC Genomics. 2015 Oct 30;16:884. doi: 10.1186/s12864-015-1965-7 (PMC4628385; doi:10.1186/s12864-015-1965-7)
Supplement: Additional file 1: — Composition of the experimental diets. (DOCX 16 kb) [file 12864_2015_1965_MOESM1_ESM.docx]

**Additional file 1:**

**Composition of the experimental diets**

|  |  | **Treatments** | |
| --- | --- | --- | --- |
| Ingredients (% of dry matter) | **Control diet** | **Diet safflower oil** | **Diet linseed oil** |
| Chopped hay | 3.3 | 3.3 | 3.3 |
| Corn silage | 28.6 | 28.6 | 28.6 |
| Hay silage | 28.5 | 28.5 | 28.5 |
| Corn grain | 23.4 | 23.4 | 23.4 |
| Soybean meal | 10.6 | 10.6 | 10.6 |
| ^1^Protein supplements | 3.7 | 3.7 | 3.7 |
| Calcium carbonate | 0.4 | 0.4 | 0.4 |
| Iodide mineral supplement | 1.5 | 1.5 | 1.5 |
| Safflower oil | - | 5 | - |
| Linseed oil | - | - | 5 |

^1^Protein supplement: 30% corn gluten feed, 30% corn distiller’s grain, 20% canola meal and 20% heat treated soybean meal.
