# Supplementary material for: Deep sequencing shows microRNA involvement in bovine mammary gland adaptation to diets supplemented with linseed oil or safflower oil
Source: BMC Genomics. 2015 Oct 30;16:884. doi: 10.1186/s12864-015-1965-7 (PMC4628385; doi:10.1186/s12864-015-1965-7)
Supplement: Additional file 2: — Library barcodes of miRNA-seq library preparation. (DOCX 16 kb) [file 12864_2015_1965_MOESM2_ESM.docx]

| Barcode | | Lane_1 | Lane_2 | Lane_3 |
| --- | --- | --- | --- | --- |
| BC1 | aagcca | 62_J-14 | 71_J-14 | 70_J-14 |
| BC2 | accgta | 62_Day+7 | 71_Day+7 | 70_Day+7 |
| BC3 | agactc | 62_Day+28 | 71_Day+28 | 70_Day+28 |
| BC4 | cagttc | 66_Day-14 | 73_Day-14 | 66_Day-14 |
| BC6 | cgtaga | 63_Day+7 | 73_Day+7 | 66_Day+7 |
| BC7 | cttgac | 63_Day+28 | 73_Day+28 | 66_Day+28 |
| BC8 | gactga | 61_Day-14 | 5355_Day-14 | 72_Day-14 |
| BC9 | gcctat | 61_Day+7 | 5355_Day+7 | 72_Day+7 |
| BC10 | gctatc | 61_Day+28 | 5355_Day+28 | 72_Day+28 |
| BC11 | ggagaa | 74_Day-14 | 2_Day-14 | 67_Day-14 |
| BC12 | ggttct | 74_Day+7 | 2_Day+7 | 67_Day+7 |
| BC13 | gtaagc | 74_Day+28 | 2_Day+28 | 67_Day+28 |

**Additional file 2:**

**Library barcodes for each miRNA-Seq library preparation**
