# Supplementary material for: Deep sequencing shows microRNA involvement in bovine mammary gland adaptation to diets supplemented with linseed oil or safflower oil
Source: BMC Genomics. 2015 Oct 30;16:884. doi: 10.1186/s12864-015-1965-7 (PMC4628385; doi:10.1186/s12864-015-1965-7)
Supplement: Additional file 5: — Top expressed miRNAs at different time points according to treatments. (DOCX 17 kb) [file 12864_2015_1965_MOESM5_ESM.docx]

**Additional file 5:**

**Top expressed miRNAs at different time points according to treatments**

|  |  | Linseed oil treatment | | | Safflower oil treatment | | |
| --- | --- | --- | --- | --- | --- | --- | --- |
| miRNA | Total reads | Day-14 | DAY+7 | DAY+28 | Day-14 | Day+7 | Day+28 |
| bta-miR-148a | 40895370 | 17.29% | 17.94% | 19.66% | 18.47% | 20.72% | 21.21% |
| bta-miR-143 | 24571780 | 12.91% | 10.68% | 10.71% | 11.60% | 10.80% | 12.47% |
| bta-miR-26a | 19562250 | 9.39% | 9.98% | 9.09% | 9.22% | 9.00% | 7.88% |
| bta-miR-30a-5p | 17767395 | 8.93% | 8.42% | 7.61% | 8.46% | 8.43% | 7.88% |
| bta-miR-10b | 12622283 | 6.11% | 5.49% | 5.77% | 6.12% | 6.05% | 5.89% |
| bta-miR-21-5p | 9323375 | 4.24% | 4.17% | 5.22% | 3.66% | 3.59% | 5.53% |
| bta-miR-99a-5p | 8878872 | 4.05% | 4.36% | 4.02% | 4.83% | 4.11% | 3.44% |
| bta-let-7a-5p | 8181342 | 3.42% | 3.99% | 4.23% | 3.42% | 3.75% | 4.22% |
| bta-miR-27b | 5262188 | 2.50% | 2.49% | 2.61% | 2.42% | 2.34% | 2.38% |
| bta-let-7f | 3640923 | 1.68% | 1.71% | 1.71% | 1.72% | 1.74% | 1.64% |

^1^The ratio refers to the total number of reads of a miRNA as compared to all reads of known miRNA detected at the same time point per treatment.
