# Supplementary figures and images for: Deep sequencing shows microRNA involvement in bovine mammary gland adaptation to diets supplemented with linseed oil or safflower oil
Source: BMC Genomics. 2015 Oct 30;16:884. doi: 10.1186/s12864-015-1965-7 (PMC4628385; doi:10.1186/s12864-015-1965-7)

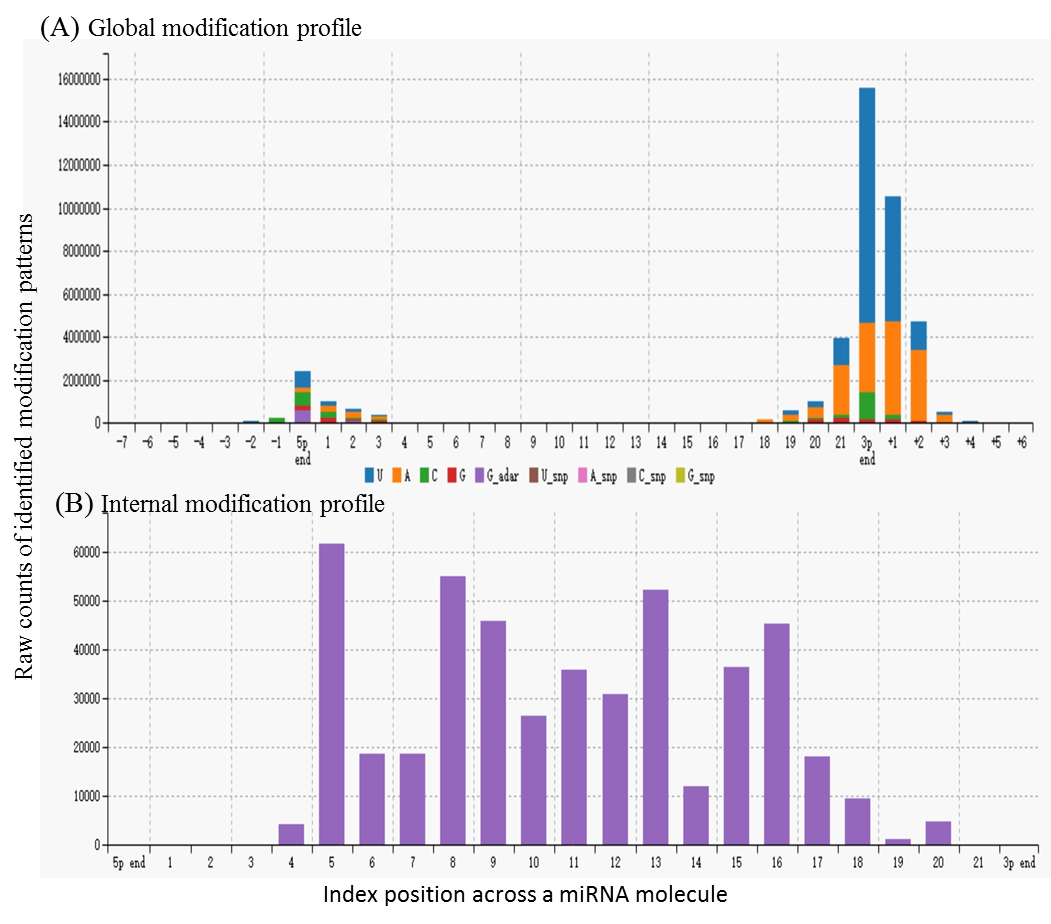

Supplement: Additional file 7: — Summary of modification analysis of dominant isomiRs that differed from consensus miRBase sequences. (A) 5’end (5p) and 3’ end (3p) modifications of isomiRs showed that single nucleotide 3’extensions are dominated by U and A modifications while 5’end modifications are much lower. (B) Internal modifications are due to deamination of adenosine to inosine by adenosine deaminases (ADAR). The start of a miRNA on the x-axis is at index ‘0’ (5' end) while its end is at index ‘22’ (3' end). Note: U, A, C and G refer to additions; G_adar refers to deamination of an adenosine to inosine by adenosine deaminases ; U_snp, A_snp, C_Snp and G_snp refer to snps which are different from the genome. (PNG 212 kb) [file 12864_2015_1965_MOESM7_ESM.png]
