# Supplementary material for: Deep sequencing shows microRNA involvement in bovine mammary gland adaptation to diets supplemented with linseed oil or safflower oil
Source: BMC Genomics. 2015 Oct 30;16:884. doi: 10.1186/s12864-015-1965-7 (PMC4628385; doi:10.1186/s12864-015-1965-7)
Supplement: Additional file 11: — Functional annotation of the targets of ( A ) up-regulated and ( B ) down regulated miRNAs in linseed oil treatment, and ( C ) up-regulated and ( D ) down-regulated miRNAs in safflower oil treatment. (PDF 290 kb) [file 12864_2015_1965_MOESM11_ESM.pdf]

Additional file 11a-d:

Functional annotation of the targets of differentially expressed miRNAs in each of the treatments

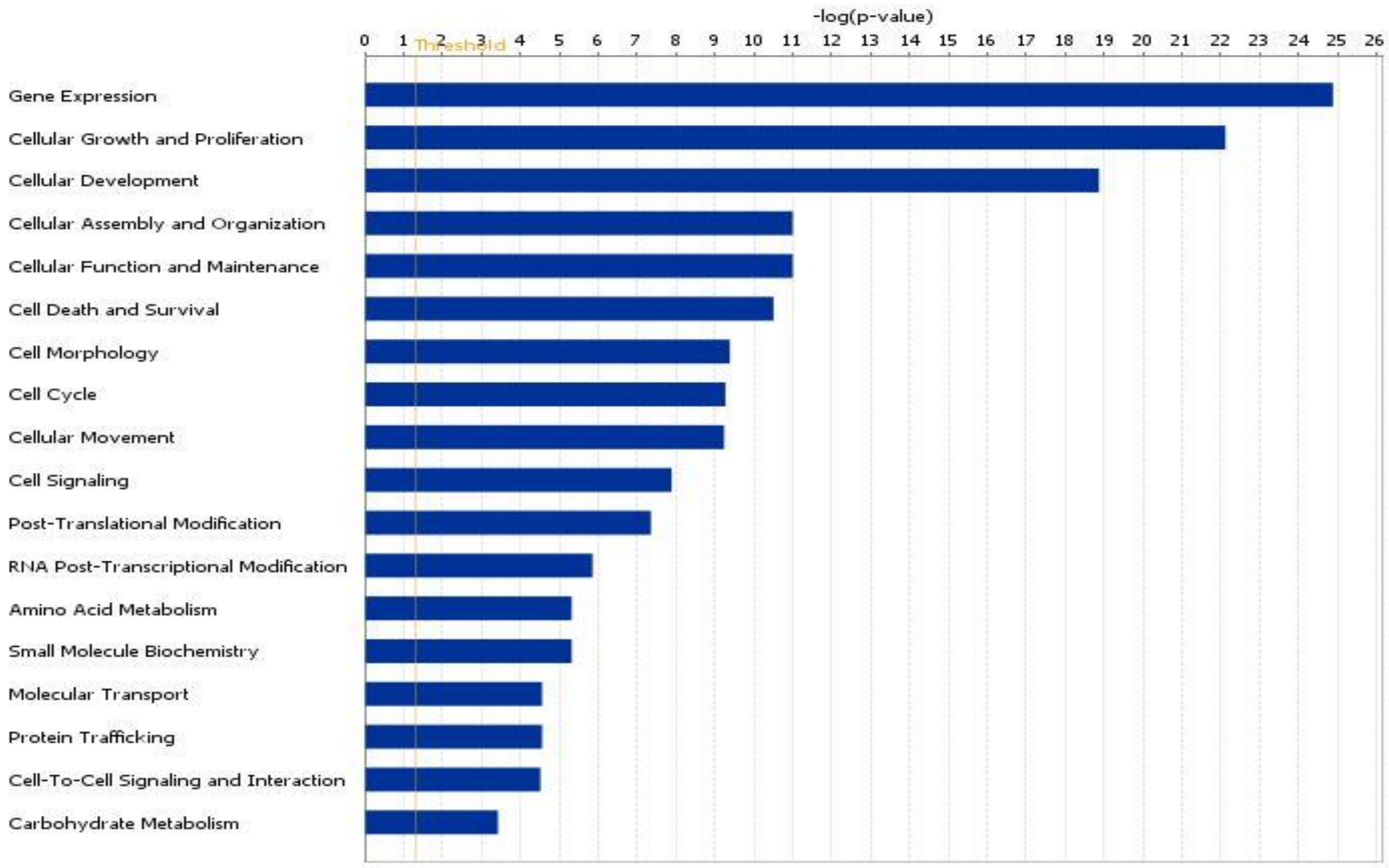

(A). Enriched functions of targets of up-regulated miRNAs in linseed group

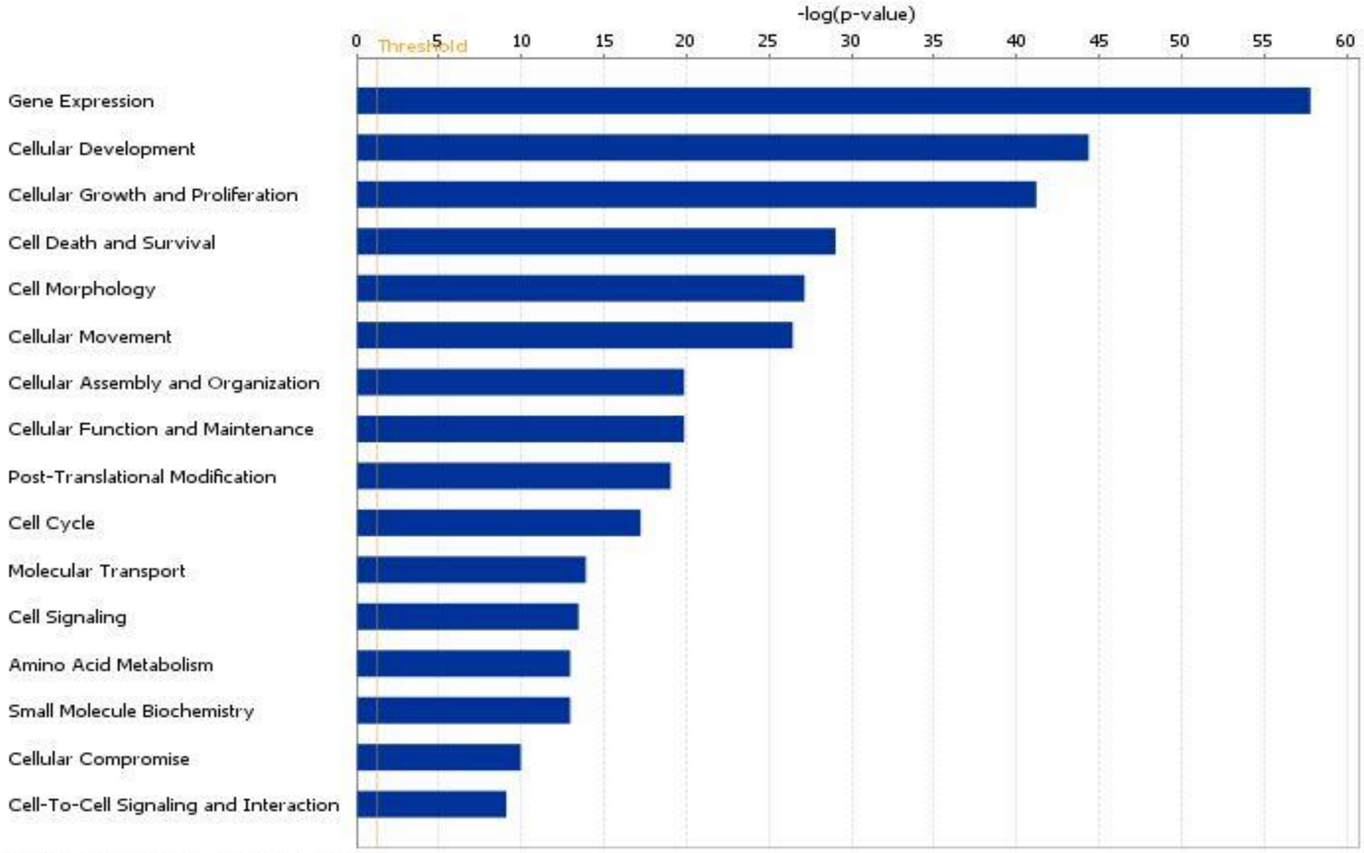

(B). Enriched functions of targets of down-regulated miRNAs in linseed group

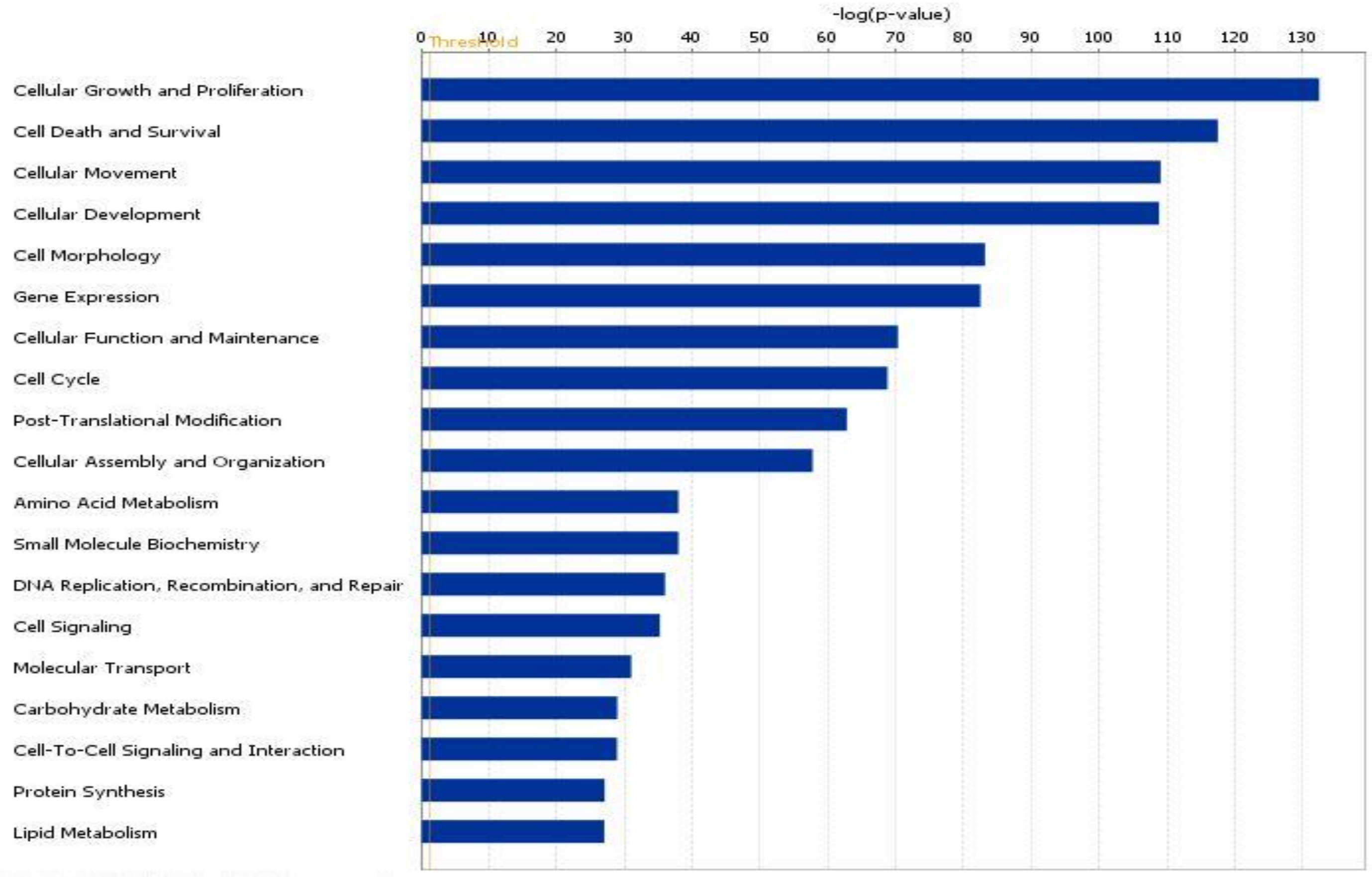

(C). Enriched functions of targets of un-regulated miRNAs in safflower group

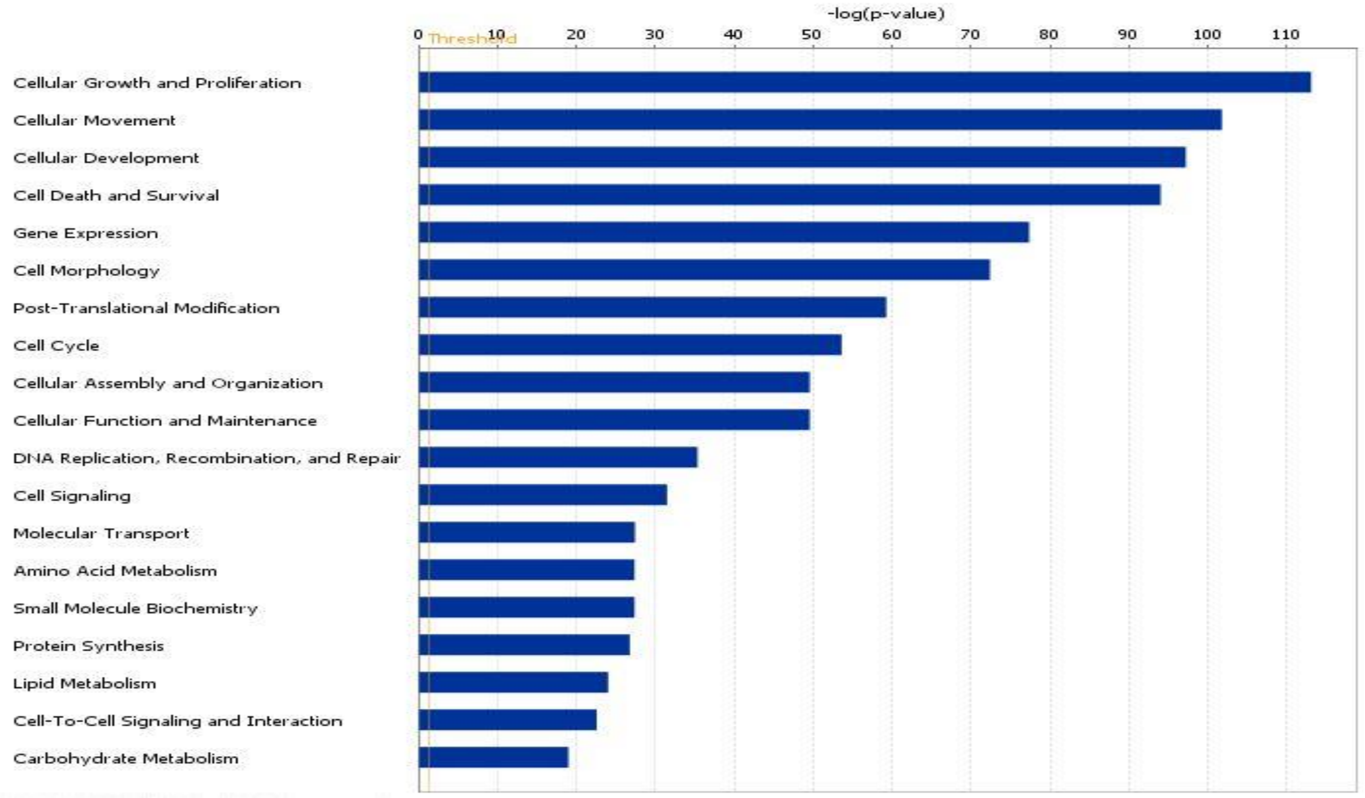

(D). **Enriched functions of targets of down-regulated miRNAs in safflower group**
